# Air Pollution and Central Nervous System Disease: A Review of the Impact of Fine Particulate Matter on Neurological Disorders

**DOI:** 10.3389/fpubh.2020.575330

**Published:** 2020-12-16

**Authors:** Hyunyoung Kim, Won-Ho Kim, Young-Youl Kim, Hyun-Young Park

**Affiliations:** ^1^Division of Allergy and Respiratory Disease Research, Department of Chronic Disease Convergence Research, Korea National Institute of Health, Cheongju-si, South Korea; ^2^Division of Cardiovascular Disease Research, Department of Chronic Disease Convergence Research, Korea National Institute of Health, Cheongju-si, South Korea; ^3^Department of Precision Medicine, Korea National Institute of Health, Cheongju-si, South Korea

**Keywords:** fine particulate matter, neurological disease, intervention, oxidative stress, epidemiological study

## Abstract

**Background:** It is widely known that the harmful effects of fine dust can cause various diseases. Research on the correlation between fine dust and health has been mainly focused on lung and cardiovascular diseases. By contrast, the effects of air pollution on the central nervous system (CNS) are not broadly recognized.

**Findings:** Air pollution can cause diverse neurological disorders as the result of inflammation of the nervous system, oxidative stress, activation of microglial cells, protein condensation, and cerebral vascular-barrier disorders, but uncertainty remains concerning the biological mechanisms by which air pollution produces neurological disease. Neuronal cell damage caused by fine dust, especially in fetuses and infants, can cause permanent brain damage or lead to neurological disease in adulthood.

**Conclusion:** It is necessary to study the air pollution–CNS disease connection with particular care and commitment. Moreover, the epidemiological and experimental study of the association between exposure to air pollution and CNS damage is critical to public health and quality of life. Here, we summarize the correlations between fine dust exposure and neurological disorders reported so far and make suggestions on the direction future research should take.

## Introduction

Air pollution is known to be the most dangerous and important environmental risk factor in the world. According to a recent report by the World Health Organization (WHO), 4.2 and 3.8 million people die early each year due to external and indoor air pollution, respectively (1).

Due to its small size, fine particulate matter (PM) lingers in the atmosphere and can easily penetrate the lungs during respiration and disseminate through the body via blood vessels, causing adverse health effects. In particular, if exposed to fine dust over a long period of time, the immune system will deteriorate rapidly, increasing the risk of various diseases such as those of the cardiovascular system and skin as well as respiratory diseases such as colds, asthma, and bronchitis.

The International Agency for Research on Cancer (IARC) has designated fine dust as a first-grade carcinogen because of its high content of heavy metals and because pollution from automobile-induced smog particles and sulfur oxides is sufficient to cause cancer.

A complex mixture of PM, gases, organic compounds, and inorganic compounds, air pollution is present both outdoors and indoors. PM is classified by aerodynamic diameter (>2.5 to <10 μm, coarse particles, PM10; <2.5 μm, fine particles, PM2.5; and <100 nm, ultrafine particulate matter, UFPM) and—given the capability of fine and UFPM to reach the brain (2)—is of special concern to brain health. In particular, the major constituents of fine dust, such as polycyclic aromatic hydrocarbons (PAH), NO_2_, and SO_2_, are reportedly major causes of cardiovascular, respiratory, neurological, eye, and skin diseases.

Evidence of air pollution's hazardous effects on the central nervous system (CNS) has been accumulating recently. Ambient air pollution is now suspected of being a neurotoxicant, and mounting evidence from human epidemiological and animal studies suggests that air pollution may negatively affect the CNS and contribute to CNS disease (2). Furthermore, unlike other organs, the lung and brain are susceptible to the direct absorption of fine PM through the nasal olfactory mucosa (3, 4).

In addition, air pollution, especially PM2.5 and nitrogen oxides (NO_x_), is known to affect the CNS, causing systemic inflammation, neuroinflammation, and oxidative stress (5–7). A growing body of literature links exposure to various air pollutants with poor brain health and an increased incidence of neurological and psychiatric disorders such as cognitive decline, dementia, anxiety, depression, schizophrenia, and attention deficit hyperactivity disorder (ADHD). Here, we summarize the available published evidence regarding associations between neurological disease and air pollution across the lifespan. We also summarize the results of epidemiological and mechanistic studies on brain diseases and suggest remedial methods and future research directions aimed at minimizing the adverse health effects of fine dust.

## Association With Neurological Disorders

### Dementia and Cognitive Function

Exposure to contaminated external air is considered an environmental risk factor that promotes brain aging. Recently, several epidemiological studies have reported that the risk of developing dementia and Alzheimer's disease is increased by exposure to fine PM (PM < 2.5 μm) (8). Despite the recent growing evidence of neurological effects of air pollution on Alzheimer's disease and associated cognitive function, no definitive conclusions on causality can be drawn, because research is as yet insufficient and uncertainty about the underlying mechanisms persists.

The role of long-term exposure to air pollution in the onset of dementia is still controversial. However, in both epidemiological and toxicological studies, exposure to air pollution seems to be associated with a decrease in cognitive function (9–12).

A steady stream of reports has linked Alzheimer's disease and exposure to ambient-air particles. For example, in *ApoE*–/– mutant mice exposed to fine dust, numbers of dopamine cells decreased by 29% compared with those of control groups breathing fresh air, indicating that fine dust has a significant impact on nerve cells (13). Moreover, continual exposure to PM2.5 neurotoxicity contributed to early decline of immediate free recall/new learning abilities, corresponding to the preclinical stage. This change was mediated by progressive atrophy of gray matter, which indicated an increased risk of Alzheimer's disease independent of cerebrovascular damage (14). Furthermore, a study in Rome found a positive association between residential exposure to NO_x_ and ozone and first hospitalizations for dementia. Moreover, exposure to PM and NO_x_ has been associated with hospitalizations for vascular dementia (8, 15, 16).

**Figure 1:**
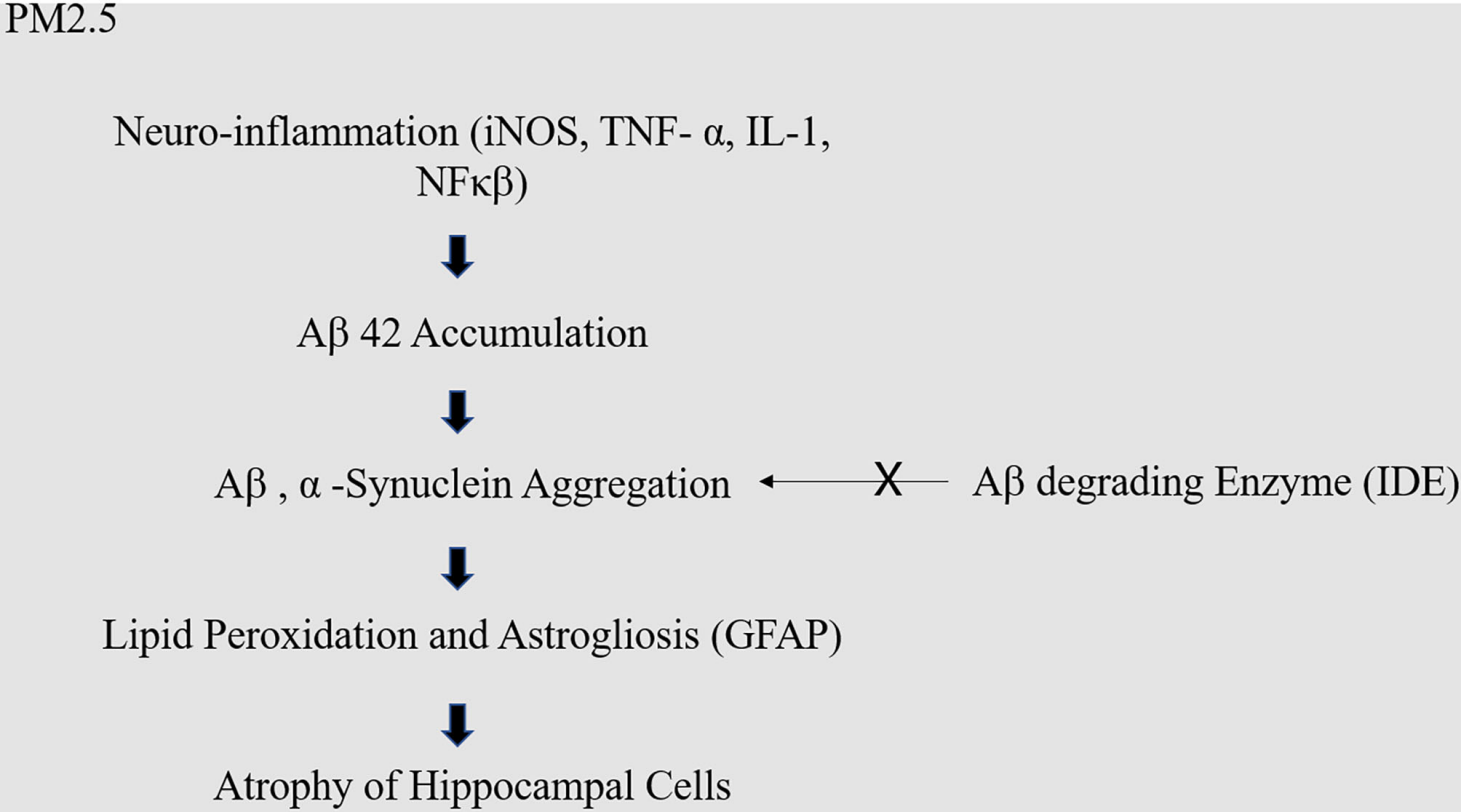
Molecular mechanism of PM2.5 in dementia. Fine dust causes neuro-inflammation, which causes excessive accumulation of amyloid. Excessively accumulated amyloid usually should be decomposed by amyloid decomposition enzyme like IDE, but the malfunction of IDE causes neuronal cell death.

Various biological pathways, such as systemic inflammation, oxidative stress, have been highlighted in attempts to explain the relationship between air pollution and brain dysfunction. It has been reported that air pollution causes metabolic abnormalities and oxidative stress in the brain. Specifically, air-pollution-induced dysfunction of the insulin signaling system reportedly reduces cognitive function and increase the risk of dementia. In particular, the neural hyper-insulinemia, glucose resistance, and amyloid beta accumulation (Aβ) was observed in genetically modified knock-out mice lacking the insulin-degrading enzyme (IDE) (17). Glucose resistance reportedly causes memory loss and hippocampal cell atrophy, and insulin resistance reduces glucose absorption in the cerebrum, thus increasing the risk of dementia (18, 19) (Figure 1). Furthermore, previous research suggests that fine dust (PM2.5) can trigger cognitive impairment and neurodegeneration through changes in the mitochondrial structure and function; specifically, PM2.5 inhalation was found to interfere with aerobic tricarboxylic acid metabolism and oxidative phosphorylation, subsequently reducing ATP production and consequently leading to hypophosphorylation of tau in the cortex of middle-aged mice. In addition, the production of excessive reactive oxygen species was implicated in this damage. These changes resulted in partial recovery after PM2.5 exposure was terminated (20).

### Stroke

It is well-known that cardiovascular risk factors play an important role in the elevation of stroke incidence observed in highly polluted urban areas. The scientific data indicate that short-term exposure to air pollution is associated with an increased risk of stroke and acute coronary syndromes such as myocardial infarction. Out-of-hospital deaths from cardiac arrest are positively associated with ambient air pollution levels (21, 22). In addition, it has frequently been reported that the toxic substances found in fine dust contribute to thrombosis in blood vessels, which increases the rate of strokes (23, 24).

The association of stroke with air pollution is more researched than that with any other neurological disorder. According to reports so far, exposure to air pollution is also correlated with subclinical diseases underlying stroke. These include systemic inflammation, oxidative stress, atherosclerosis, thrombosis, and arrhythmia (23).

Several studies have implicated continuous exposure to PM2.5 in the causation of stroke (25–27). A recent study reported that NO_2_, the major component of air pollution, is one of the main causes of stroke. Moreover, short-term exposure to SO_2_, NO_2_, and PM10 was significantly associated with increased risk of ischemic stroke (28, 29). These toxic components are associated with elevation of the daily maximum heart rate, heart block frequency, and atrial fibrillation (30). Long-term residential exposure to locally emitted black carbon from traffic exhaust was associated with ischemic stroke incidence (31).

**Figure 2:**
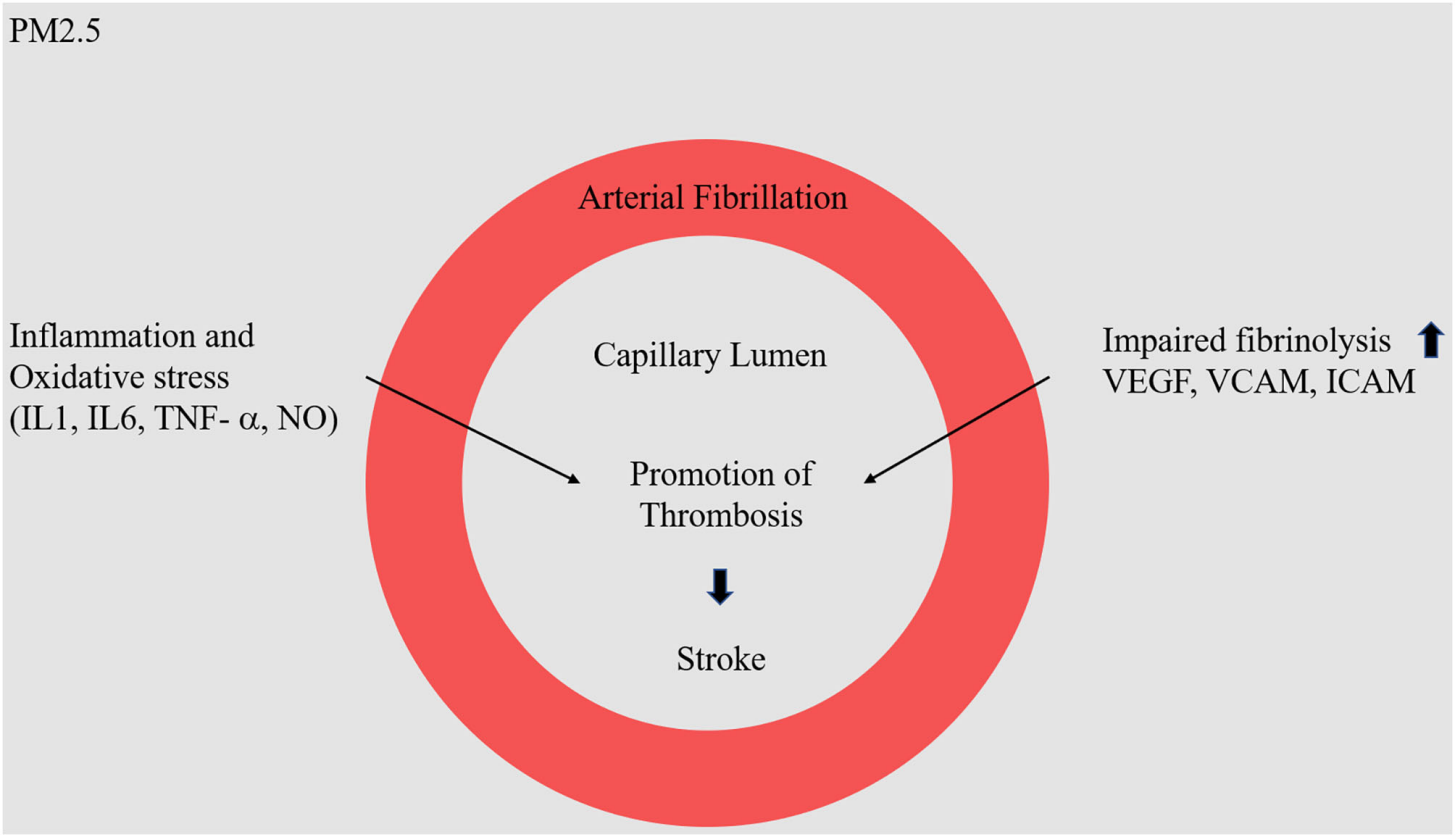
Molecular mechanism of PM2.5 in stroke. Fine dust (NO^−^, O_3_) enters the blood vessel, it will cause inflammatory and oxidative stress reactions. In particular, as the expression of VEGF, VCAM, and ICAM protein increases, there is a high probability of causing problems in the physical barrier in blood vessels, causing arterial fibrillation.

Many papers report on the correlation between air pollution and stroke mortality, but little is known about the specific subtypes of PM2.5 that are the most hazardous in this regard. Therefore, correlating types of pollutants with stroke incidence is very important for the assessment of health impacts (32) (Figure 2).

### Depression

Depression is one of the most common mental health problems worldwide. According to a WHO report, more than 300 million people are living with depression, with an increase of 18% from 2005 to 2015 (33).

To date, many epidemiological papers have been published correlating air pollution with depression. However, this correlation remains controversial. The risk of depression is significantly increased with long-term exposure to PM2.5 and short-term exposure to PM10, NO_2_, SO_2_, and CO, but not exposure to O_3_ (34–36). On the other hand, other reports suggest that O_3_ is strongly associated with depression in women (37, 38). In addition, short-term exposure to air pollution did not produce clear pathological alterations in mood in a healthy elderly population (39).

In a study of mice exposed to air pollution, expression of the gene underlying the oxidative stress response was attenuated when the mice were injected with an inhibitor of cortisol production. However, when they were injected with cortisol itself, the oxidative stress-causing gene was activated (40). These results suggest that cortisol level is a key player in the effect of air pollution on depression. The brain is highly sensitive to glucocorticoids, such as cortisol, which is a stress hormone with important roles in cognition and depression. The response to stress starts in the CNS and acts via the hypothalamus and pituitary gland to increase the secretion of cortisol from the adrenal cortex (17, 41). Recent epidemiological research revealed the correlation between air pollution and cortisol levels, and that NO_2_, PM2.5, and PM10 were reportedly associated with high wake-up cortisol (42, 43). When HPA is activated by air pollution and glucocorticoid binds to the glucocorticoid receptor (GR), the associated neurotrophic factor and the genes associated with inflammatory reactions, apoptosis, and metabolism are expressed, resulting in impaired neurogenesis, neurotoxicity, and glial cell reactivity. This eventually leads to cognitive decline, dementia, and depression (17, 40, 44).

A clear demonstration of a causal relationship between air pollution (or some toxic component thereof) and depression remains elusive, and further research on this question is needed.

### Multiple Sclerosis

Multiple sclerosis (MS) is a chronic disease of the central nervous system that is caused by inflammatory reactions or neurodegeneration. This prevalence of MS is ~2.5 million. While the underlying etiology of autoimmune diseases remains unclear, environmental and genetic factors have been implicated as major contributors. According to recent studies, about 40−70% of all autoimmune diseases can be explained by environmental factors, including air pollution, sunlight, ultraviolet rays, vitamin D, Epstein-Barr virus (EBV), smoking, and secondhand smoke (45–47).

Several studies have investigated the correlation between air pollution and MS, and although the results have been conflicting, they can be largely summarized in terms of three hypotheses. First, that particulate matter is absorbed into the lungs through the respiratory tract, inducing pulmonary/systemic inflammation and the activation of T cells, which then secrete proinflammatory cytokines in the brain; second, that particulate matter absorbed directly through the olfactory bulb may induce inflammatory reactions in the brain; and third, that epigenetic mutations caused by fine dust influence the onset of MS (48).

According to a recent analysis of the CSF in healthy children, the levels of IL-2, IL-6, and MIF (MIF) increased in children exposed to high concentrations of air pollution. IL-2 specifically has been established as a target of MS treatment (49). The reported induction of inflammation and oxidative stress in the brain by air pollution may affect brain autoimmunity, which may consequently influence MS disease or development (50).

While research on smoking and other environmental factors has considered their roles in DNA modification and the prevalence of MS, studies on fine dust have tended to focus more on the correlation between oxidative stress and inflammation or MS than on DNA modification (50, 51).

According to epidemiological studies, fine dust can be a risk factor for MS (52–54). The results of epidemiological surveys conducted in Italy showed that that air pollution may be an additional environmental risk factor that might contribute to the pathophysiology of MS (54).

In addition, a study that examined the changes in brain MRI findings of MS patients due to PM10 exposure confirmed that PM10 caused MS favoring inflammatory reactions (6). However, few studies have reported contrary findings. An examination of the long-term effects of air pollution revealed that that PM2.5, NO_2_, and O_3_ did not affect MS prevalence, and other cohort studies showed no correlation between air pollution and MS risk (6, 55). Since epidemiological findings about the correlation between fine dust and MS disease are still contextual, more epidemiological, and cohort studies are needed.

### Schizophrenia

Schizophrenia is a chronic and severe mental disorder affecting more than 21 million people worldwide. It is generally accepted that mental illness is more prevalent among urban residents than rural (56, 57). A meta-analysis of the regional prevalence of mental illness found a 30% higher rate of mental illness among urban dwellers than their non-urban counterparts; the rate was 21% higher for anxiety disorders and 39% higher for mood disorders (58). Furthermore, a time series study of a possible link between outpatient visits because of schizophrenia and exposure to short-term air pollution reported that short-term exposure to air pollution (PM10, SO_2_, and NO_2_) was associated with an increased risk of daily outpatient visits because of schizophrenia (59).

Several studies have been published in recent years on the correlation between the prevalence of mental illness and levels of fine airborne PM (7, 60, 61). According to a recent study, teenagers exposed to four types of air pollutant—NO_2_, NO_x_, PM2.5, and PM10—have a 60% higher rate of mental illness than do those not so exposed (60, 62). In addition, a Chinese study that examined the number of outpatient visits by patients with schizophrenia and its correlation with air pollution (PM_10_, SO_2_, and NO_2_) found that outpatient visits increased significantly if patients were exposed to outside air pollution for only a short time (59, 63). Moreover, the severity of schizophrenia symptoms was exacerbated by high ambient PM2.5 concentrations in patients over 65 years of age (64). These findings show that, if continuously exposed to fine dust, all age groups are affected by an exacerbation and increased prevalence of schizophrenia. However, despite many epidemiological reports showing a link between fine dust and schizophrenia, few reports address the question of how fine dust might affect the development of schizophrenia mechanistically.

### ADHD

Many studies over the past decade report that air pollution has a negative impact on child brain development, especially on the triggers of ADHD and autism spectrum disorder (ASD) (65). Several genetic and environmental factors are suspected of affecting the ASD phenotype, including air pollution, exposure to pesticides, maternal infections, inflammatory conditions, dietary factors, and administration of antibiotics during pregnancy.

The association of fine dust with ADHD was investigated by a Danish cohort study that ran from 1992 to 2007. The results showed that the higher the concentration of PM2.5 and NO_2_ in residential areas, the higher the incidence of ADHD; NO_2_ had a particularly clear association (66). Recent reports suggest that exposure to PM late in pregnancy significantly decreases the area of the corpus callosum (CC) of the brain, and a reduction of the CC area of 50 mm^3^ leads to significantly higher hyperactivity sub-scores (67).

Few studies have investigated a direct correlation between fine dust and ADHD onset. Although some epidemiological data may be correlated, further research on the exact mechanism is needed.

### Neurodevelopment

It is known that several chemicals concealed in the living environment act as major toxins in neural development. In particular, airborne fine PM contains toxic substances such as lead, methylmercury, arsenic, polychlorinated biphenyls, and toluene, and these have neurotoxic activity and are thought to affect nerve development (68).

**Figure 3:**
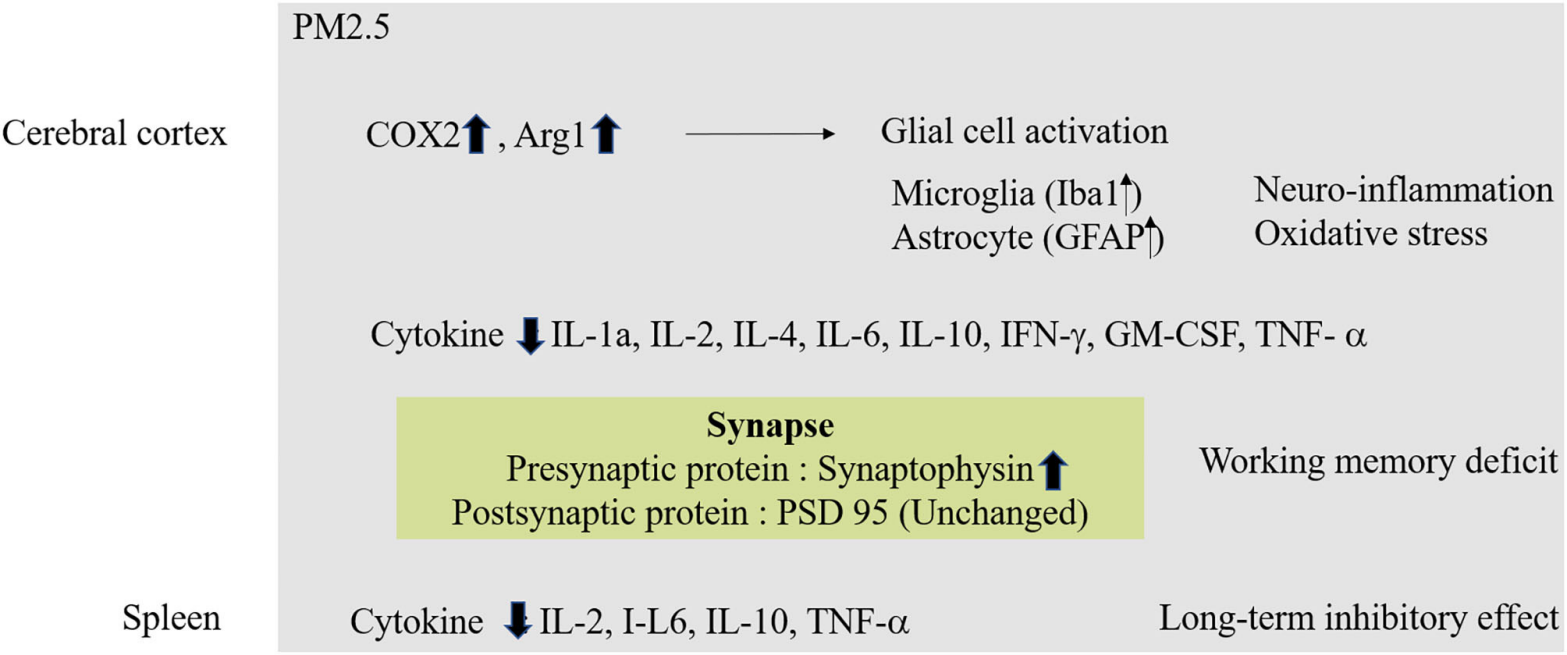
Molecular mechanism of PM2.5 in Neurodevelopment. In case of exposure to fine dust in fetal conditions, oxidative stress and inflammatory reactions occur in the cerebral cortex. Also, abnormal protein expression in synapses can affect working memory. Exposure to fine dust in fetal conditions also affects other organs, which can lead to long-term memory deficiencies.

Continuous exposure to high levels of ambient air pollution during pregnancy can lead to a greater negative effect on brain development than exposure during childhood. Because the structure of the brain is established during fetal life, exposure to substances originating in air pollution *in utero* can lead to permanent brain damage or to cognitive impairment in old age (69). A cohort study reported that exposure to PM2.5 and O_3_ at an early stage of development was associated with neurodevelopmental delay (70, 71) (Figure 3).

A recent study investigating the mechanistic link between fine dust and neurogenesis reported that human nerve cells treated with ultrafine dust showed significant increases in levels of the messenger RNA specifying metallothioneins 1A and 1F. The researchers also found that air pollution affected DNA methylation, which in turn affected protein expression at synapses. Another study found that exposure to fine dust in pregnancy not only lowers IQ, but also decreases levels of brain-derived neurotrophic factor, leading to lower gray-matter volumes in the brain (14). Further research on the molecular mechanism of the link between fine dust exposure and neurodevelopment seems necessary (72, 73).

The evidence of an association between ambient air pollution and neurological disease in general is summarized in Table 1.

**Table 1:** Evidence of association between ambient air pollution and neurological disease.

**Diseases**	**Subjects**	**Study design**	**Exposure level (component)**	**Result**	**References**
Dementia	9,817,806	Meta-analysis	PM_2.5_	Long-term exposure PM2.5 accreted time to first hospitalization for dementia.	(11)
	130,978	Retrospective cohort study	PM2.5 and PM2.5 from traffic	Positive association between residential levels of air pollution across London and being diagnosed with dementia.	(12)
	95,690	Prospective cohort study	PM2.5, O_3_	Long term exposure to PM2.5 and O_3_ above the current US EPA standards are associated with increased risk of AD.	(8)
	2,066,639	Prospective cohort study	PM2.5, NO_2_	15, 813 cases of dementia (or 6.1% of total cases) were attributable to elevated air pollution.	(16)
	19,409	Prospective cohort study	PM2.5, PM10	Rate of cognitive decline was significantly higher in women with highest level of exposure to PM2.5 compared to lowest level.	(10)
	20,150	Prospective cohort study	PM2.5	Exposure to PM2.5 was not associated with incident cognitive impairment, even when analysis was run on participants with more than 12 months of exposure data.	(74)
	2,867	Prospective cohort study	PM2.5, PM10 (w/or w/o Traffic)	Exposure to PM2.5 and PM10 was associated with memory decline in participants.	(75)
	130,978	Retrospective cohort study	PM2.5, NO_2_, O_3_	Increased risk of dementia with increased exposure to PM2.5 and NO_2_. Decreased risk with greater exposure to O_3_.	(12)
	2,066,639	Prospective cohort study	PM2.5, NO_2_, O_3_	PM2.5 and NO_2_ is associated increased risk of dementia. But, increased exposure to O_3_ was not associated with incident dementia.	(16)
	5,116	Case-Control design	PM2.5, O_3_	Presence of at least one *APOE4* allele was associated with faster decline for air pollution. O_3_ exposure effects were not significant in cognitively impaired but associated with faster decline for all exposure.	(76)
	243,611	Prospective cohort study	PM2.5, NO_2_	Living closer to a roadway was associated with increased risk of dementia.	(16)
	29,547	Case-Control design	NO_2_, CO	Highest levels of NO_2_ and CO exposure was significantly associated with increased risk of dementia.	(77)
Stroke	65,893 (Postmenopausal women)	Prospective cohort study	PM2.5	Stroke incidence increased by 35% and stroke deaths increased by 83% after exposure to PM2.5.	(22, 78)
	124,614	Prospective cohort	PM2.5	Stroke incidence increased by 19% after exposure to PM2.5.	(26)
	836,557	Prospective cohort study (Patient)	SO_2_	Stroke incidence increased by 4% after exposure to SO_2_.	(29)
	24,066	Prospective cohort study	PM2.5	Stroke hospitalization increased by 3.49% after the exposure to PM2.5.	(27)
	9,941	Retrospective cohort	PM10, NO_2_	Stroke mortality increased by 49% for PM10 and 144% for NO_2_.	(79)
	99,446	Meta-analysis	PM2.5	Overall stroke incidence increased by 19%. Increased risk was observed even at concentrations that met the European Union standard of 25μg/m^3^.	(80)
	379	Retrospective cross-sectional study	PM10, PM2.5, NO_2_, O_3_, SO_2_	Air pollution levels correlate with ischemic stroke admission.	(81)
	2,640,000	Case-Control design	PM2.5	Air temperature influences air pollution and hospital admission for stroke.	(82)
	10,663	Case-Control design	NO_2_	NO_2_ levels during the cold season is associated with increase stroke admissions.	(83)
	3,362	Prospective cohort study (Time-series design)	NO, SO_2_, CO, organic carbon	Each component associated with hemorrhagic stroke.	(84)
	26,210	Case-Control design	O_3_	O_3_ exposure associated with increased stroke hospitalization risk.	(85)
Depression	118,602 DD (Depressive Disorder)	Case-Control design	PM2.5, NO_2_, SO_2_, O_3_	The ozone was more strongly associated with depressive symptoms in warm season. But there was no association with PM2.5, SO_2_, or NO_2_.	(35)
	19,646 DD	Case-Control design	PM2.5, PM10	The PM2.5 and PM10 were associated with incidence of depression, and aging is a susceptibility factor.	(86)
	27,047 DD	Time-series study	PM2.5, NO_2_, SO_2_, O_3_	The PM2.5, NO_2_, SO_2_, and CO had significant association with depression in warm season.	(38)
	4,985 DD	Case-Control design	PM10, NO_2_, SO_2_, O_3_	The PM10, NO_2_, SO_2_, and CO had significant association with depression.	(36)
	680 DD	Case-Control design	O_3_	The O_3_ was strongly associated with depression in women.	(37)
	973 DD	Prospective cohort study	PM2.5	The PM2.5 is associated with incidence of depression and chronic disease is a susceptibility factor.	(87)
Schizophrenia	943, 027	Meta-analysis	Urban vs. suburban	Natural environments during childhood may be important for schizophrenia prevention.	(57)
	10,947 MDs (Mental Disorder)	Case-Control design (Time-series)	PM2.5, PM10, PMc	PM exposure might be an important trigger of hospitalizations for MDs.	(61)
	1,193 SP (Schizophrenia patients)	Cross-sectional Study	PM2.5	Ambient PM2.5 concentration was associated with exacerbation of schizophrenia.	(64)
	34,865 SP	Case-Control design (Time-series)	PM2.5, SO_2_, NO_2_	Ambient air pollution (PM10, SO_2_, NO_2_) can be associated with increased risk of daily outpatient visits for schizophrenia.	(59)
	11,373 MDs	Case-Control design (Time-series)	NO_2_	Short-term exposure to NO_2_ may be associated with increased schizophrenia hospital admissions.	(62)
	2,232 (Children)	Cross-sectional study	NOx, PM2.5, PM10	Air pollution exposure-particularly NO_2_ and NO_x_-was associated with increased odds of adolescent psychotic experiences, which partly explained the association between urban residency and adolescent psychotic experiences.	(60)
	3,469 MDs	Case-Control design (Time-series)	PM10, SO_2_, NO_2_	It significantly increased the risk of schizophrenia episode in subjects who were male, aged 20–59, farmers, and workers.	(63)

*MD, Mental Disorder Patient; DD, Depressive Disorder Patient; SP, Schizophrenia Patient*.

## Pathophysiological Mechanisms in the Nervous System

Air-polluting substances could potentially reach the brain via the olfactory tract, the gastro-intestinal tract/vagus nerve, or the blood-brain barrier (BBB). The most direct of these routes is direct absorption into the brain through the olfactory bulb. Although the biological mechanisms by which fine dust affects brain diseases are unclear, it has been reported that oxidative stress and inflammatory reactions are the two major processes by which air pollution exerts its toxic systemic and CNS effects (4, 88, 89).

In addition to the direct input of fine dust into the brain, there is an indirect absorption path into the brain through respiratory intake. In this case, the indirect input causes systematic inflammation and leads to circulating cytokines that pass through the BBB. Astrocytes in the brain respond to the neuroinflammation and oxidative stress by eventually inducing cell death (89).

In an animal study, oxidative stress was examined as a function of brain region by measuring lipid peroxidation after exposure to diesel-exhaust particulates (DEP). Elevations in a number of pro-inflammatory cytokines (IL-1α, IL-6, IL-10, IL-13, IL-5, and TNF-α) were observed, particularly in the olfactory bulb and hippocampus, but DEP caused an increase in lipid peroxidation in all brain regions, with levels of inflammatory cytokines increasing and levels of the pleiotropic cytokine IL-9 decreasing (4, 90, 91).

According to a recent study, the neurotoxicity induced by DEP can be mediated by the inflammatory reaction of microglial cells. DEP is neurotoxic only to neurons co-cultured with microglial cells and not to neurons alone. Further, these effects are reversible when the microglial cells involved are treated with pioglitazone, an antagonist of the peroxisome proliferator-activated receptor-gamma (PPAR-γ) (41). PPARs are known to be ligand-activated transcription factors, regulating genes essential for various metabolic processes and cell differentiation, but also exerting anti-inflammatory actions after brain injury or in neurodegenerative diseases (92).

Oxidative stress produced by DEP is also closely related to neurodegenerative disease. In a whole-body exposure study in mice, Aβ_1−42_ and tau protein, which are markers for Alzheimer's disease, showed increased levels in the frontal and temporal lobes. Moreover, α-synuclein, the causative protein of Parkinson's disease, showed increased levels in the midbrain. These two brain regions are closely related to schizophrenia, depression, dementia, and Parkinson's disease (4).

**Figure 4:**
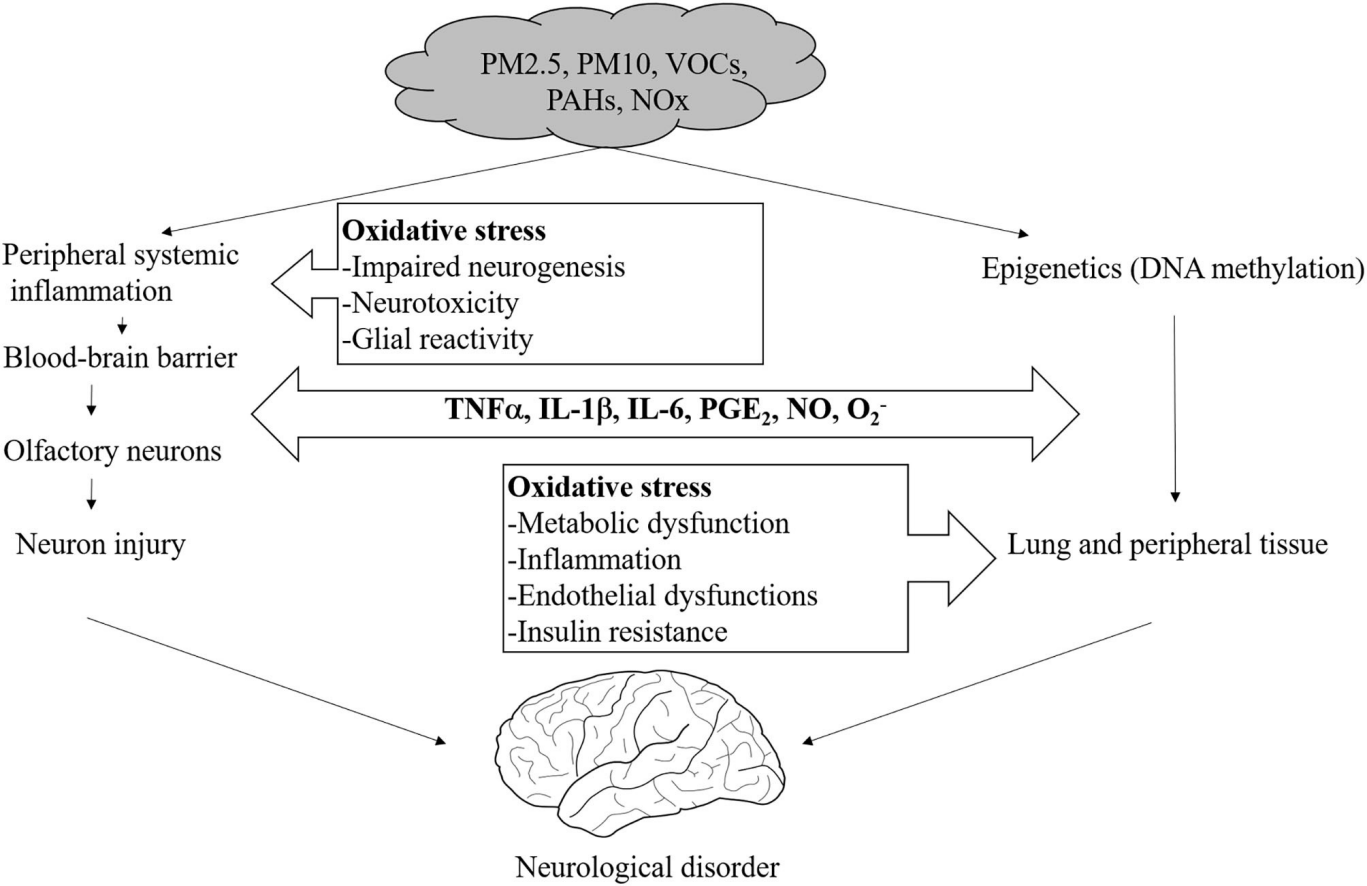
Proposed mechanisms of direct and systemic pollutant-induced oxidative stress in the brain.

In addition, recent studies have shown that air pollutants alter BBB function. For example, aluminum nanoparticles have been reported to injure endothelial cells and damage the BBB (93). Human exposure to air pollution results in endothelial-cell damage in the cerebral vasculature, with increases in the expression of intercellular adhesion molecules (ICAMs) and vascular cell adhesion molecules (VCAMs) (94). A rat whole-brain *in vitro* study showed that exposure to PM causes the production of cytokines and reactive oxygen species, a decrease in the expression of various tight-junction proteins, and changes in the intracellular signaling pathway that governs the expression and function of the xenobiotic transporter (95) (Figure 4).

Various omics analyses were conducted to identify the physiological mechanisms of various diseases caused by fine dust. Genetic studies reported changes in DNA additives, telomere length, and mitochondria DNA (mtDNA) content caused by fine dust (PM2.5, PAH, NO_2_) (96–98). Transcriptomics analysis revealed a reduction in the expression of BDNF and CYP1A1 (99), and epigenetics analysis showed changes in DNA methylation [mtDNA, lone interspersed nuclear element 1(LINE-1), leptin (LEP) promotor] and miRNA expression (mir-21, miR-146a, and miR-22) (99–101). Proteomics analysis found an increase in AHH activity and decreases in pyruvate kinase, MT, and GST activity (102–104).

### Interventions to Reduce the Impact of Air Pollution on Neurodegenerative Disease

To date, the most common intervention in neurological diseases caused by air pollution is to minimize exposure to ambient air contaminants.

Several recent reports suggest that dietary supplementation with antioxidants such as certain vitamins reduces the harmful effects of fine dust. In particular, it was reported that vitamins C and E prevent oxidative damage from exposure to O_3_ and PM2.5, and unsaturated fatty acids such as fish oil and olive oil have the effect of mitigating cardiovascular reactions to short-term exposure to air pollutants (105). It was also reported that the effects on the cardiovascular system of exposure to fine dust are reduced by dietary B vitamins (106). Moreover, PM2.5 causes methylation of the genes related to energy metabolism in mitochondria, changes that are reduced by dietary B vitamins (107). However, these studies had small sample sizes and did not examine the chemical components of the fine particulates. Further research is needed on whether antioxidant foods can mitigate the general effects of fine dust.

To mitigate diseases caused by environmental pollution, the complexities of environmental, dietary, and sociological parameters related to the interaction of genes and epigenetics will have to be considered in addition to segmental research; hence, future research requires high-level biological coupling.

Using the complex Exposome paradigm, the European Research Group recently proved that contaminated industrial sites are directly linked to health problems. This study is thought to be an important example of future research methodology for investigations into diseases attributable because of environmental pollution (108, 109).

Fine dust has varied effects on neurological diseases because its composition varies from source to source. Therefore, no one intervention method can be appropriate in all circumstances. At present, minimizing exposure remains the only way to minimize damage. General medical policy is as follows: Prevent fine-dust damage to the bedridden patient with neurological disease, respiratory disease, etc. by managing blood pressure, diabetes, and obesity, and by counseling the patient to refrain from outdoor activities and strenuous exercise. Wearing masks is also recommended in cases of severe exposure to fine dust, and if the disease worsens, the patient is advised to visit a hospital.

To date, research on the health effects of fine PM has accounted for most short-term exposure studies; several papers have described fine-dust effects on acute respiratory diseases. However, interventional studies are challenging because most neurological diseases aggravated by fine PM would involve long-term exposure.

Therefore, to mitigate health damage caused by fine dust, personal efforts such as improvement in indoor air quality should be encouraged, and public health efforts should be made to reduce fine dust in the air generally. Until now, most of the papers on general intervention studies are written from a preventive medical and epidemiological point of view, but few studies have investigated mitigation based on biological mechanisms. Due to the seriousness of the fine-dust problem that has become known in recent years, we hope that many interventional studies based on biological mechanisms will be conducted in the near future.

## Future Directions

As many studies have shown, air pollution has a deleterious effect on neurological disorders. However, to prove this relationship clearly and find a way to mitigate the effects of air pollution, further systematic studies with improved precision seem necessary.

The rates of diseases such as dementia and depression continue to rise year by year in Korea, where fine dust levels are above average compared with other Organization for Economic Cooperation and Development countries. Of course, many factors are involved beyond the effects of air pollution. However, multiple studies have found an epidemiological link between air pollution and certain neurological diseases. Therefore, a major interventional study will be required in the future to investigate a possible causal relationship.

## Limitations and Conclusion

This review has certain important limitations. First, this is not a systematic review, and therefore there were no specific criteria for selecting the included articles, and results were not analyzed using statistical methods. In addition, the scope of our review of air pollution and cerebral neuropathy was wide, and individual diseases have not been discussed in-depth. Finally, previously known harmful mechanisms, such as oxidative stress and inflammation, and new or disease-specific mechanisms have not been covered in detail. Additional work will be required to determine the precise impact of air pollution on each neurological disorder.

It is time to go beyond epidemiological studies and first verify, then elucidate, the effects of fine dust on various diseases through research into biological mechanisms. Most of the mechanistic studies so far have focused on two basic disease-causing processes: oxidative stress and inflammation. Research on any mechanisms specific to fine dust will also be necessary. In addition, there is a need for evidence-based treatment approaches to the problem of exacerbation of underlying disease by fine dust.

Epidemiological studies are also required to analyze the causal relationship of fine dust to neurological diseases with respect to composition and source. In addition, researchers should seek ways to minimize the impact of fine dust on brain diseases through biological mechanistic studies.

## Author Contributions

HK was a major contributor in writing the manuscript. W-HK participated in the data and interpretation of the part of the Stroke. Y-YK participated in the collection and interpretation of epidemiological data. H-YP was a contributor in the composition and writing of the whole idea and thesis. All authors made contributions to the interpretation of data and revising the manuscript. All authors read and approved the final manuscript.

## Conflict of Interest

The authors declare that the research was conducted in the absence of any commercial or financial relationships that could be construed as a potential conflict of interest.
